# Scoping Review of Socio-Ecological Factors Contributing to Sleep Health Disparities in Children with Autism Spectrum Disorder

**DOI:** 10.1007/s10803-025-06807-x

**Published:** 2025-04-05

**Authors:** Megan L. Wenzell, Carolyn E. Ievers-Landis, Sehyun Kim, Samantha DeSimio, Mandy Neudecker, Siobhan Aaron, Kelly Wierenga, Meng Miao, Ariel A. Williamson

**Affiliations:** 1 Frances Payne Bolton School of Nursing, Case Western Reserve University, 9501 Euclid Ave, Cleveland, OH 44106 USA; 2 Reserve University School of Medicine, University Hospitals Rainbow Babies & Children’s Hospital, 11100 Euclid Ave, Cleveland, OH 44106-6038 USA; 3 Case Western Reserve University School of Medicine, 9501 Euclid Ave, Cleveland, OH 44106 USA; 4 University Rainbow Babies and Children’s Hospital, 2101 Adelbert Rd, Cleveland, OH 44106 USA; 5 Frances Payne Bolton, School of Nursing, Case Western Reserve University, 9501 Euclid Ave, Cleveland, OH 44106 USA; 6 School of Nursing, Indiana University, 600 Barnhill Dr, 46202 Indianapolis, IN USA; 7 The Ballmer Institute for Children’s Behavioral Health, University of Oregon, 2800 NE Liberty St, 97211 Portland, OR USA

**Keywords:** Autism spectrum disorder, Adolescent, Child, Health disparities, Sleep health

## Abstract

Given the high prevalence of sleep problems in children with autism spectrum disorder (ASD), there is a critical need to examine how sleep problems may be exacerbated for children exposed to social and environmental adversity. Guided by the socio-ecological model, this review aimed to evaluate factors linked to sleep health disparities (SHDs) in children with ASD, determine possible gaps/limitations in existing literature, and identify possible solutions. A scoping review was selected to ascertain what is known about SHDs in ASD. Four databases identified articles from 2004 to 2023. Included articles were those conducted in children with ASD that focused on sleep and examined socio-ecological factors (i.e., individual, family, neighborhood and socio-cultural) possibly contributing to SHDs. 41 studies were extracted; 31 (75.6%) focused on individual factors, 27 (65.9%) focused on family factors, 11 (26.8%) focused on neighborhood and/or socio-cultural factors; 3 (7.3%) focused on factors across all three socio-ecological levels. Six studies included interventions that found improvements in child sleep, behavior, and quality of life. Representation of racial and ethnic minoritized groups was limited across studies. Most studies focused on individual child factors associated with sleep problems, with less research focused on family factors, and very few studies examining broader neighborhood and socio-cultural factors. Only about half of studies reported race and ethnicity data, with sparse representation of racial and ethnic minoritized children and families overall. These findings highlight the need for future research on modifiable socio-ecological factors to guide equitable sleep interventions for children with ASD.

**Supplementary Information:**

The online version contains supplementary material available at 10.1007/s10803-025-06807-x.

Autism spectrum disorder (ASD) affects as many as 1 in 36 school-age children in the United States (American Psychiatric Association, 2013; Maenner et al., 2023). Approximately 53 to 78% of children with ASD aged 5 to 12 years have sleep problems, which is two times greater than the prevalence of sleep problems in general pediatrics (Couturier et al., 2005; Goodlin-Jones et al., 2008; Krakowiak et al., 2008). Parents of children with ASD most commonly report insomnia, defined as persistent difficulty falling or staying sleep and/or early morning awakenings (American Academy of Sleep Medicine, AASM, 2014; Liu et al., 2006). In children with ASD, behavioral features such as insistence on sameness, noncompliance with parental requests, and hyperactivity may contribute to increased risk of sleep problems (Goldman et al., 2009). If left untreated, sleep problems can contribute to daytime disruptive behavior and impaired learning for children with ASD (Bianca et al., 2022; Hirata et al., 2016). Longer-term possible detrimental impacts of sleep problems include obesity, diabetes, and cardiovascular disease (Colten & Altevogt, 2006).

In September 2023, the National Institute on Minority Health and Health Disparities (NIMHD) identified people with disabilities as a health disparity population, or a population experiencing differences in one or more health outcomes due to historical and ongoing exposure to adverse social and environmental factors (Calman et al., 2023; NIMHD, 2023). Other NIMHD-defined health disparity populations include those of racial and ethnic minoritized backgrounds, lower socioeconomic status (SES) backgrounds, and those living in rural communities. To date, research has primarily focused on sleep health disparities (SHDs), or differences in dimensions of sleep health (sleep-related behaviors, duration, regularity, satisfaction, quality, and timing; Buysse, 2014; Meltzer et al., 2021) that adversely and consistently affect children of racial and ethnic minoritized and/or lower-SES backgrounds (Billings et al., 2021; Blanc et al., 2023; Lupini & Williamson, 2023). Much of this work has applied Bronfenbrenner’s socio-ecological framework (1992) to identify the individual child (microsystem), family (mesosystem), and neighborhood and broader socio-cultural factors (macrosystem) that likely interact to produce these SHDs (Meltzer et al., 2021). These factors may be proximally or distally linked to sleep health in children with ASD. Individual factors, for example, disruptive behavior or co-existing health conditions (e.g., epilepsy, attention-deficit/hyperactivity disorder) (Liu et al., 2006; Won et al., 2019), and family factors, such as bedtime parenting practices and parent health-related quality of life (Iwamoto et al., 2023; Levin & Scher, 2016), may have a more direct impact on child sleep problems when compared to neighborhood cohesion and safety (Mayne et al., 2021). Although sleep problems are well-documented among children with ASD, comprehensive reviews of the socio-ecological factors that likely contribute to SHDs in children with ASD are lacking. For instance, children with ASD tend to experience more co-existing conditions (e.g., hyperactivity, anxiety) that could contribute to or perpetuate sleep problems (Johnson et al., 2018; Sikora et al., 2012). Caregivers may also seek treatment for other ASD symptoms, delaying sleep problem identification and management.

In addition, few studies have taken an intersectional lens to identify the extent of SHDs among children with ASD that are also part of additional health disparity populations (Crenshaw, 1989). For example, children with ASD who also identify as being of a racial and ethnic minoritized background may experience racism as well as disability-related discrimination, both of which could contribute to poor health outcomes and SHDs (Jackson et al., 2020). SES-related disparities could also exacerbate limited access to behavioral sleep care for children with ASD. However, to our knowledge, this issue has not been explored in children with ASD. Existing studies on health disparities in ASD have focused on provision of resources, access to early and high-quality care, quality of health insurance, exposure to adverse childhood experiences, and other sociodemographic factors (Karpur et al., 2019; Mandell et al., 2009; Parikh et al., 2018).

Using a socio-ecological framework to better understand SHDs in children with ASD could help to enhance the acceptability, effectiveness, and equity of interventions to promote sleep health and address sleep problems. In general pediatrics, socioeconomic factors at multiple socio-ecological levels, including maternal education, family income and resources (e.g., availability of an individual bed or room), and neighborhood SES have been associated with child sleep problems (El-Sheikh et al., 2013; Spilsbury et al., 2006). Other studies have identified additional individual child, family, and broader socio-cultural factors linked to sleep outcomes (Covington et al., 2021; Newton et al., 2020). Just as incorporating these factors into sleep intervention strategies and delivery methods could benefit general pediatric populations, understanding these factors in the context of SHDs in children with ASD could inform the development and/or enhancement of behavioral sleep treatment for children with ASD. This review aimed to (1) examine socio-ecological factors linked to SHDs, as well as the inclusion of other health disparity populations, (2) determine possible gaps/limitations in existing literature, and (3) identify various ways in which existing behavioral sleep interventions could be tailored for mitigating SHDs among children with ASD.

## Method

### Search Strategy

A scoping review was selected to broadly ascertain what is known about socio-ecological factors contributing to SHDs in children with ASD (Munn et al., 2018). In October 2023, we searched Elsevier Embase, EBSCO Cumulative Index to Nursing and Allied Health Literature (CINAHL), Ovid Medline, Ovid PsychINFO, and hand searched relevant articles with the help of an experienced librarian. The search included database-controlled vocabulary and text word searching with Boolean operators. Key search terms (see **Appendix A**) included ASD, sleep, social determinants of health, and health inequities. Time filters were not applied to the search. Duplicates were removed with remaining articles uploaded into *Covidence Systematic Review Software* (2024). Following study protocols, two authors reviewed title and abstracts to ensure they met the following criteria: (1) included children with ASD, (2) focused key search terms, and (3) examined socio-ecological factors contributing to SHDs. Articles were excluded if the article focused solely on parents of children with ASD, were not peer reviewed (e.g., dissertations, registered trials), or were not published in English. Conference abstracts were excluded. Articles that met the inclusion criteria were moved to a separate Covidence library for full-text analysis. Two authors completed the full-text analysis to identify the final set of articles. Articles published between 2004 and 2023 were included.

Titles and abstracts of articles were reviewed via the key search terms and uploaded to the Covidence database. Two reviewers followed protocols to screen the articles in the following steps: (1) Title and abstracts and (2) Full text review, and three reviewers completed the (3) Extraction. A consensus agreement with a third rater on the research team was established to resolve conflict.

### Identification and Abstraction of Relevant Studies

**Fig. 1:**
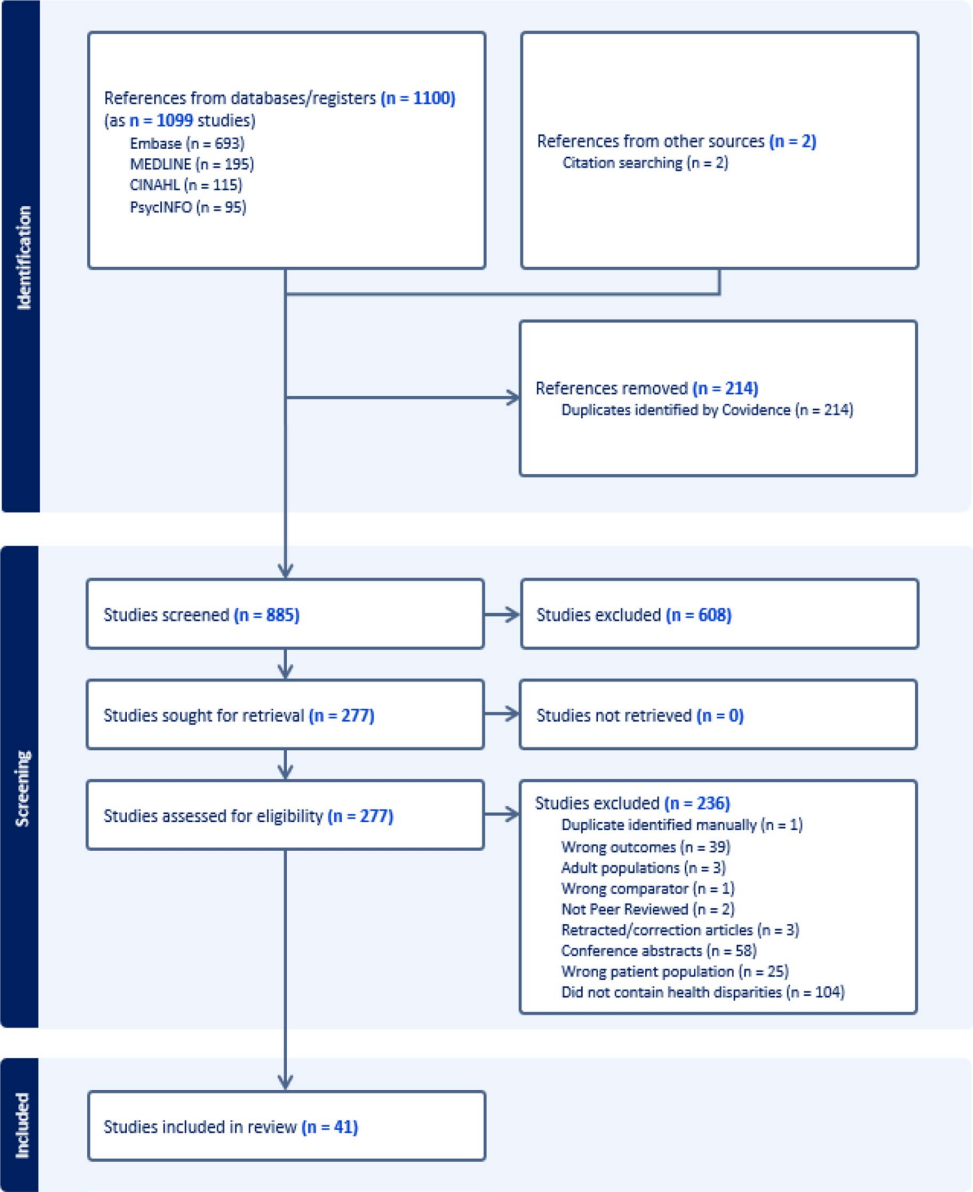
PRISMA flowchart of article selection process

The PRISMA flowchart is presented in Fig. 1. The combined searches resulted in 1099 articles. Covidence removed 214 duplicate articles; 885 articles were screened; and 41 were included in the final review. Sample size, study design, sociodemographic information (age, race and ethnicity, location), and SES indicators (caregiver educational attainment, family income, household structure, neighborhood-level SES, etc.) were extracted from included articles to characterize study samples. Of note, race and ethnicity are socio-political constructs and not indicators of biological or genetic differences (Boyd et al., 2020; Flanagin et al., 2021) and were included to identify the extent to which NIMHD-identified health disparity populations were included in this work. Findings from the 41 studies were categorized to reflect individual child, family, and neighborhood and broader socio-cultural factors, according to a socio-ecological framework by Bronfenbrenner and Ceci (1994).

## Results

Table 1 displays the characteristics of the studies included.

**Table 1:** Characteristics of included studies

Study	Sample Size ^a^; Sociodemographic characteristics: Ages Studied (Yrs.); Sex (%, *n*); Race and Ethnicity (%, *n*); Location	Socioeconomic Status (SES) Indicators	Socio-ecological Factors Addressed/Main Findings
1– Baker et al. (2023)	*N* = 397; Mean Age = 6.45 *±* 3.70 yrs., Range = 4–15; Age at wave 1 = 4–5 yrs.; 77.1% Male (*n* = 306); Race/Ethnicity NR; Australia	*Maternal Highest Education*: 40.4% Advanced Diploma (*n* = 154), 36.2% Tertiary (*n* = 138), 23.4% HS or less (*n* = 89); *Maternal Employment*: 53.5%, Employed (*n* = 204), 43.6% Not in labour force (*n* = 166), 2.9% Unemployed (*n* = 11); *Family Structure*: 84.1% Married (*n* = 323), 15.9% Single parent (*n* = 61); *SEIFA Australia* = 1000.3 *±* 74.6	*Family Factors*: • Maternal psychological distress when children were aged 4–5 years predicted child sleep problems at age 6–7 years • Child sleep problems when children were aged 12–13 years predicted greater maternal psychological distress when children were aged 14–15 years
2– Bin Eid et al. (2022)	*N* = 116 ^a^; *Saudi Arabia (n* *= 81)*: Mean Age = 8.18 *±* 1.56 yrs., Range = 5–12 yrs.; 82.7% Male (*n* = 67); Race/Ethnicity NR; *United Kingdom (n* *= 35)*: Mean Age = 9.21 *±* 1.97 yrs.; 74.3% Male (*n* = 26); Race/Ethnicity NR	*Maternal Highest Education (ASD)*: 0% Postgraduate degree, 53% Undergraduate degree (*n* = 43), 23.5% GCSE A-Levels Vocational Level 3 (*n* = 19), 16.1% GCSE O-Levels Vocational Level 2 (*n =* 13), 7.4% No Qualification (*n* = 6) *Paternal Highest Education (ASD)*: 22.2% Postgraduate (*n* = 18), 39.5% Undergraduate degree (*n* = 32), 18.5% GCSE A-Levels Vocational Level 3 (*n* = 15), 12.4% GCSE O-Levels Vocational Level 2 (*n* = 10), 7.4% No Qualification (*n* = 6)	*Neighborhood and Socio-cultural Factors*: • Overall, children with ASD in Saudi Arabia experience poorer sleep than children with ASD in the United Kingdom • Children with ASD in Saudi Arabia had shorter sleep duration than children with ASD in the United Kingdom • Children with ASD in the United Kingdom had more sleep anxiety and more parasomnias than children with ASD in Saudi Arabia *Family Factors*: • Children with ASD in Saudi Arabia were exposed to more screen time than children with ASD in the United Kingdom • Children with ASD in the United Kingdom had more regular mealtimes than children with ASD in Saudi Arabia
3– Broder-Fingert et al. (2014)	*N* = 2,976 ^a^; Age range = 2–20 yrs.; *ASD (n* *= 2*,*075)*: 79.5% Male (*n* = 1,650); 80.7% White (*n* = 1,670), 7.4% Hispanic/Latino (*n* = 153), 5.5% Black (*n* = 114), 6.4% Other (*n* = 132); *Asperger’s (n* *= 901)*: 78.7% Male (*n* = 709); 81.3% White (*n* = 733), 3.6% Black (*n* = 33), 6.4% Hispanic/Latino (*n* = 58), 8.6% Other (*n* = 77); US	*Insurance (ASD)*: 44.9% Private (*n* = 931), 55.1% Public (*n* = 1,144); *Insurance (Asperger’s)*: 55.2% Private (*n* = 497), 44.8% Public (*n* = 403)	*Neighborhood and Socio-cultural Factors*: • Among children with ASD, those of older age, with public insurance, and with a co-occurring sleep disorder had a higher odds of being overweight and/or obese
4– Bruni et al. (2022)	*N* = 111; Age range = 1–18 yrs.; 83.8% Male (*n* = 93); Race/Ethnicity NR; Italy	*Caregiver Highest Education*: 38.7% (*n* = 43), 51.4% HS (*n* = 57), 8.1% Middle school (*n* = 9), 1.8% Elementary school (*n* = 2). *Family Income*: 77.5% Middle income (*n* = 86), 18.9% Low income (*n* = 21), 3.6% High income (*n* = 4)	*Neighborhood and Socio-cultural Factors*: • Bedtime, rise time, and sleep duration changed during pandemic • Difficulty falling asleep, bedtime anxiety, sleep terrors, and daytime sleepiness increased during the pandemic • Daily screen time increased during the pandemic, and caregivers identified that having fewer obligations and extracurricular activities contributed to more screen time, which in turn impacted sleep
**5**– Davenport et al. (2021)	*N* = 1; Mean Age = 7 yrs. 4 months; Male; Biracial; US	NR	*Individual Factors*: • After the intervention, sleep onset latency decreased and sleep efficiency improved • Improved sleep was associated with decreased daytime disruptive behavior *Neighborhood and Socio-cultural Factors*: • The authors speculated that telehealth could enhance access, benefiting underserved communities and those with financial disadvantage
6– Delahaye et al. (2014)	*N* = 86; 69.5% ASD, 25% PDD-NOS, 5% Asperger’s; Mean Age = 7.18 *±* NR yrs., Range = 4–12 yrs.; 83.7% Male (*n* = 72); Race/Ethnicity NR; US	NR	*Individual Factors*: • Sleep habits were correlated with physical and psychosocial QoL • Physical QoL was correlated with daytime sleepiness, parasomnias, sleep duration, and sleep anxiety • Physical QoL was *not* correlated with bedtime resistance, night wakings, sleep-disordered breathing, or sleep onset delay • Psychosocial QoL was correlated with parasomnias, sleep-disordered breathing, sleep duration, sleep onset delay, and sleep anxiety • Psychosocial QoL was not correlated with bedtime resistance, daytime sleepiness, and night wakings
7– Durán-Pacheco et al. (2023)	*N* = 3,150; Median Age = 9.0 yrs., Range = 3–17 yrs.; 80.1% Male (*n* = 2,507); 5.2% Non-White/Hispanic (*n* = 163), 16.1% Non-White/Non-Hispanic (*n* = 507), 10.0% White/Hispanic (*n* = 315), 68.7% White/Non-Hispanic (*n* = 2,165); US	*Household Income*: 36.1% <$50,000 annually (*n* = 1,137), 32.8% $50,000–$99,999 (*n* = 1,034), 27.2% *≥*$100,000 (*n* = 856), 3.9% Prefer not to answer (*n* = 122); *Caregiver Employment*: 43.3% Full-time (*n* = 1,360), 29.4% Homemaker (*n* = 924), 18.7% Part-time (*n* = 588), 2.8% Unemployed (*n* = 89), 2.4% Student (*n* = 75), 0.7% Retired (*n* = 23), 2.5% Other (*n* = 79); *Household Structure*: 28.6% 2–3 members (*n* = 898), 39.2% 4 members (*n* = 1,233), 32.2% ≥ 5 members (*n* = 1,014); *Family Structure*: 80% has a partner (*n* = 2,505)	*Individual Factors*: • Various factors were associated with worse sleep quality in children with ASD, including older age, female sex, medication use for ASD, and longer time since ASD diagnosis • After controlling for demographic factors, children who eloped, experienced mental comorbidities, or a greater impact of ASD core symptoms had poor sleep quality *Family Factors*: • Lower household income and larger household size (*≥* 5) were linked to worse sleep quality • After controlling for demographic factors, caregiver impression of ASD severity was linked to poor child sleep quality *Neighborhood and Socio-cultural Factors*: • Living in the northeast US region was associated with worse child sleep quality compared to the west
8– Elkhatib Smidt et al. (2022)	*N* = 681 ^a^; Mean Age = 12.1 *±* 3.5 yrs., Range = 6–12 yrs.; 77.8% Male (*n* = 530); 75.9% White (*n* = 517), 8.8% *≥* Two Races (*n* = 60), 2.1% Other Race (*n* = 14), 0.3% Pacific Islander (*n* = 2), 7.1% Black (*n* = 48), 4.6% Asian (*n* = 31), 1.3% American Indian/Alaskan Native (*n* = 9); 12.0% Hispanic (*n* = 82); US	*Caregiver Education*: 54.5% College degree or higher (*n* = 371), 28.9% Associate degree (*n* = 197), 14.1% HS (*n* = 96), 2.5% Less than HS (*n* = 17)	*Individual Factors*: • After adjusting for covariates, being physically active 1–3, 4–6, and 7 days a week is associated with increased odds of sufficient sleep duration for both ASD and non-ASD children • Probability of sufficient sleep duration was greater for non-autistic children than children with severe ASD • Females had a significantly lower positive effect of physical activity on sleep, compared to non-ASD females • No age-group differences in associations between physical activity and sleep duration were found
9– Elkhatib Smidt et al. (2020)	*N* = 4,636; Mean Age = 6.6 *±* 3.5 yrs., Range = 2-17.5 yrs.; 83.7% Male; 4.5% Asian, 7.2% African American or Black Canadian, 80.9% White, 7.5% Other/multiracial; 9.2% Hispanic; US/Canada	*Caregiver Education*: 4.3% < HS, 16.8% Finished HS, 32.8% Some college, 27.9% Bachelor’s degree, 18.2% Postgraduate degree	*Individual Factors*: • Younger age, Hispanic ethnicity, higher IQ, and a diagnosis of ASD were associated with poorer sleep habits • Poorer skills in daily living, socialization, and communication are associated with poorer sleep habits. *Family Factors*: • Lower primary caregiver education level was associated with greater sleep problems in children with ASD
10– Ezell et al. (2016)	*N* = 5,787; Mean Age = 6.2 *±* 3.4 yrs., Range = 1.5–17.6 yrs.; *Nonadoptees with ASD (n* *= 5*,*624)*: Mean Age = 6.2 *±* 3.4 yrs.; 84.1% Male; 80.5% White, 19.5% Non-White; 8.3% Hispanic/Latino; *Adoptees with ASD (* *= 163)*: Mean Age = 7.9 *±* 3.8 yrs.; 72.4% Male; 74.2% White, 25.8% Non-White; 10.4% Hispanic/Latino; US	NR	*Individual/Family Factors*: • There was no association between difficulty staying asleep/restlessness or difficulty falling asleep in adopted and nonadopted children with ASD • Adopted children had significantly more sleep problems and sleep medications than the non-adopted group
11– Galli et al. (2022)	*N* = 100; Mean Age = 5.56 *±* 2.28 yrs., Range = 2.06–12.68 yrs.; 79% Male (*n* = 79); Race/Ethnicity NR; Italy	*Maternal Highest Education*: 1.0% Elementary school (*n* = 1), 19.0% Middle school (*n* = 19), 55.0% Vocational or HS (*n* = 55), 20.0% Bachelor’s degree (*n* = 20), 5.0% Master’s and/or PhD (*n* = 5); *Maternal Employment Status*: 51.0% Unemployed (*n* = 51), 49.0% Employed (*n* = 49); *Paternal Highest Education*: 1.0% Elementary school (*n* = 1), 23.0% Middle school (*n* = 23), 57.0% Vocational or HS (*n* = 57), 15.0% Bachelor’s degree (*n* = 15), 49.0% Master’s and/or PhD (*n* = 4); *Paternal Employment Status*: 95.0% Employed (*n* = 95), 5.0% Unemployed (*n* = 5); *Marital Status*: 87.0% Married (*n* = 87), 11.0% Single (*n* = 11), 2.0% Divorced: (*n* = 2); *Household SES (Avg. Hollingshead score)*: 30.4 (SD = 11.6, range 8–66)	*Individual Factors*: • Sleep disorders were associated with developmental delay, emotional and behavioral problems, and the absence of strategies used to induce sleep after night wakings • No association was found between sleep problems and epilepsy or between melatonin and resolution of insomnia *Family Factors*: • No association was found between sleep disturbance and family stress or family variables
12– Garcia et al. (2020)	*N* = 49; Mean Age = 12.4 *±* 3 yrs., Range 8–17 yrs.; 78% Male (*n* = 36); 64% White (*n* = 25); US	NR	*Individual Factors* • Children who met sleep duration recommendations were significantly younger and had higher physical activity levels than children who did not meet the recommendations • A significantly greater number of children who were “Caucasian” met the criteria for sleep efficiency compared to children from minority groups • Children who met both sleep duration and efficiency criteria had fewer sedentary minutes per day compared to those who only met the sleep efficiency criteria *Family Factors*: • Children with recommended sleep duration were more likely to meet screen time recommendations
13– Han et al. (2022)	—	—	*Individual Factors*: • Sleep problems were correlated with increased clinical symptomatology (e.g., anxiety, depression), and externalizing symptoms (e.g., aggression, hostility), adaptive and executive functioning, and physical health (e.g., healthy eating, physical activity) • Sleep problems were not significantly associated with age across studies *Family Factors*: • Sleep problems were correlated with family factors, such as SES and family history of medical, neurodevelopmental, and psychiatric conditions
14– Herrmann (2016)	—	—	*Individual Factors*: • Children with comorbid hyperactivity, emotional problems, and conduct disorder are at an increased risk of sleep disorders • Developmental regression in children with ASD is linked to higher prevalence of sleep disorders *Family Factors* • Child sleep problems may be linked to maternal sleep, stress and health
15– Hodge et al. (2013)	*N* = 90 ^a^; Mean Age = 7.49 *±* NR yrs., Range = 4–12 yrs.; 78.9% Male (*n* = 71); 43% White, 18% Hispanic, 17% Black or African American, 4% Asian, 1% Middle Eastern, 1% Native American, 16% Other/mixed ethnicity; US	NR	*Family Factors* • Maternal stress and maternal sleep were each mediating variables through which child sleep problems affected maternal mental health
16– Iwamoto et al. (2023)	*N* = 26; Mean Age = 4.45 *±* 0.79 yrs., Range = 3–5 yrs.; 73.1% Male (*n* = 19); 23.1% White, non-Hispanic/Latino (*n* = 6), 50% White, Hispanic/Latino (*n* = 13), 11.5% Asian, non-Hispanic/Latino (*n* = 3), 7.7% Asian, Hispanic/Latino (*n* = 2), 3.8% Pacific Islander, non-Hispanic/Latino (*n* = 1), 7.7% Native American & White, Hispanic/Latino (*n* = 2); US	*Caregiver Education*: 11.5% Graduate degree (*n* = 3), 26.9% Bachelor’s degree (*n* = 7), 23.0% Some college (*n* = 6), 15.4% Technical or associate degree (*n* = 4), 19.2% HS or less (*n* = 5); *Household Income (Annual)*: 26.9% >$90k (*n* = 7), 23.0% $70k to < $90k (*n* = 6), 15.4% < $30k (*n* = 4), 15.4% $30k to <$50k (*n* = 4), 11.5% $50k to <$70k (*n* = 3)	*Individual Factors*: • Shorter sleep duration was associated with greater disruptive behavior *Family Factors*: • Child behavior was related to parental stress after controlling for sleep duration
17– Jeon et al. (2023)	*N* = 68 ^a^; Age Range = 6-12.92 yrs.; *United Kingdom ASD (n* *= 35)*: Mean Age = 9.21 *±* 1.97 yrs.; 74.3% Male (*n* = 26); Race/Ethnicity NR; 45.7% CARS-2 standard (*n* = 16), 54.3% high functioning (*n* = 19); *South Korea ASD (n* *= 33)*: Mean Age = 8.27 *±* 1.89 yrs., 78.8% Male (*n* = 26); Race/Ethnicity NR	*United Kingdom ASD*: *Family Members*: 4.14 *±* 0.97 *Maternal Highest Education*: 34.3% Postgraduate (*n* = 12), 40.0% Undergraduate (*n* = 14), 25.7% A-levels, vocational level 3 or equiv. (*n* = 9) *Paternal Highest Education*: 15.4% Postgraduate (*n* = 4), 50% Undergraduate (*n* = 13), 23.1% A-levels, vocational level 3 or equiv. (*n* = *6*), 7.7% GCSE/O-Level Grade A^*^C, vocational level 2 or equiv. (*n* = 2), 3.8% No qualification (*n* = 1) *South Korea ASD*: *Family Members*: 3.91 *±* 0.84 *Maternal Highest Education*: 21.9% Postgraduate (*n* = 7), 68.8% Undergraduate (*n* = 22), 9.4% A-levels, vocational level 3 or equiv. (*n* = *3*) *Paternal Highest Education*: 21.9% Postgraduate (*n* = 7), 65.6% Undergraduate (*n* = 21), 9.4% A-levels, vocational level 3 or equiv. (*n* = *3*), 3.1% GCSE/O-Level Grade A^*^C, vocational level 2 or equiv. (*n* = 1)	*Neighborhood and Socio-cultural Factors*: • Significant effects were found in sleep duration and parasomnias in between children with ASD in the UK compared to Korea • Children with ASD in the UK had an earlier bedtime, waketime, longer time in bed, and sleep time than children in Korea with ASD • Children with ASD in Korea had later bedtimes and shorter sleep latency
**18–** Johnson et al. (2023)	*N* = 74; Mean Age = 2–7 yrs.; *Sleep Parent Training* (*n* *= 36*): Mean Age = 3.6 *±* 1.4 yrs.; 81% Male (*n* = 29); 8.3% Black (*n* = 3), 5.6% Asian/Pacific Islander (*n* = 2), 81% White (*n* = 29), 5.6% More than one race/other (*n* = 2); *Sleep Parent Education* (*n* *= 38*): Mean Age = 3.8 *±* 1.4 yrs.; 87% Male (*n* = 33); 13% Black (*n* = 5), 2.6% Asian/Pacific Islander (*n* = 1), 63% White (*n* = 24), 18% more than one race/other (*n* = 7), 2.6% unknown (*n* = 1); US	*Sleep Parent Training*: *Caregiver’s Highest Education*: 39% Advanced graduate/professional degree (*n* = 14), 36% College graduate (*n* = 13), 14% Some college/post-HS or 2-Year degree (*n* = 5), 11% HS graduate/GED (*n* = 4); *Child Living Arrangement*: 97% Parental home (*n* = 35), 2.8% Other relative (*n* = 1) *Sleep Parent Education*: *Caregiver’s Highest Education*: 55% Advanced graduate/professional degree (*n* = 21), 24% College graduate (*n* = 9), 11% Some college or post-HS or 2-year degree (*n* = 4), 11% HS graduate/GED (*n* = 4); *Child Living Arrangement*: 97% Parental home (*n* = 37), 2.6% Other relative (*n* = 1)	*Individual Factors*: • Children who received the telehealth sleep intervention had greater improvements in bedtime and sleep disturbance • No differences in irritability scores were found across groups *Family Factors*: • Children who received the sleep intervention vs. the control group did not have any differences in parental stress • Children who received the sleep intervention had improvements in parental self-efficacy scores compared to controls *Neighborhood and Socio-cultural Factors*: • Telehealth may reduce healthcare costs and enhance access, benefiting those who are underserved and those with lower incomes • 61% lived in rural areas vs. 39% lived in urban areas
19– Leader et al. (2022)	*N* = 118; Mean Age = 9.55 *±* 3.74 yrs.; 78% Male (*n* = 92); Race/Ethnicity NR; Ireland	NR	*Individual Factors*: • Daytime sleepiness was the highest reported sleep disturbance in children age 3–18 years • Children with ASD + ADHD had more sleep problems and bedtime resistance than children with ASD only • ADHD symptoms add to the complexity of ASD and sleep problems
20– Leader et al. (2022a, b)	*N* = 9; Mean Age = 9.75 *±* 2.53 yrs., Range = 7–14 yrs.; 67% Male (*n* = 6); Race/Ethnicity NR; Ireland	*Marital Status*: 8% single (*n* = 1), 67% married (*n* = 8), 17% divorced (*n* = 2); *Parent Educational Level*: 33% Secondary school (*n* = 4), 67% Tertiary education (*n* = 8)	*Family Factors*: • Parental depressive symptoms may be linked to child sleep problems, specifically wake after sleep onset
21– Levin and Scher (2016)	*N* = 66; *ASD (n* *= 35)*: Mean Age = 3.27 *±* 0.44 yrs., Range = 2.4-4 yrs.; 71.4% Male (*n* = 25); Race/Ethnicity NR; *TD (n* *= 31)*: Mean Age = 36.23 *±* 5.75 months, Range = 25–48 months; 48.4% Male (*n* = 15); Race/Ethnicity NR; Israel	*ASD: Caregiver education (Avg. yrs.)*: 13.9 (SD = 3.0). Yrs. range: 12–18; *Marital Status*: 91.2% Married (*n* = 31), 8.8% Divorced (*n* = 3); *TD: Caregiver education (Avg. yrs.)*: 15.0 (SD = 2.2). Yrs. range: 12–18; *Marital Status*: 93.5% Married (*n* = 29), 6.5% Divorced (*n* = 2)	*Family Factors*: • Child sleep problem scores accounted for 39–50% of the variance in maternal stress • Problematic maternal sleep-related cognitions, related to doubts about parenting competence and limit setting difficulties, were linked to greater child sleep problems
22– Lewis et al. (2023)	—	—	*Neighborhood and Socio-cultural Factors*: • Children were vulnerable to disrupted sleep during COVID-19 due to environmental stressors • Sleep problems in ASD worsened during COVID-19: reduced sleep duration and quality, difficulty falling asleep, frequent night awakenings, and difficulty waking
**23–** MacDonald et al. (2021)	*N* = 43; *Study 1 (n* *= 10)*: Mean Age = 8.1 *±* 2.5 yrs., Range = 2–10 yrs.; 70% Male (*n* = 7), Race/Ethnicity NR; *Study 2 (n* *= 33)*: Mean Age = 6.2 *±* 2.7 yrs., Range = 2–12 yrs.; 75.8% Male (*n* = 25), Race/Ethnicity NR; US	*Family SES*: *Study 1*, Avg. Hollingshead score = 36.4 (SD = 11.4) *Study 2*, Avg. Hollingshead score = 40.0 (SD = 14.8)	*Individual Factors*: • Modest improvements in sleep and behavior were reported *Family Factors*: • Parents valued accessible research- and community-based sleep interventions and working with familiar therapists *Neighborhood and Socio-cultural Factors*: • Community therapists implemented a sleep program
**24–** Malow et al. (2014)	*N* = 80; *Individual Education (n* *= 47)*: Mean Age = 5.6 *±* 2.6 yrs.; 83% Male (*n* = 39); 80% White (*n* = 37); *Group Education (n* *= 33)*: Mean Age = 5.9 *±* 2.8 yrs.; 76% Male (*n* = 25); 84% White (*n* = 26); US	*Family SES*: *Individual*: Avg. Hollingshead score = 44.3 (SD = 13.5) *Group*: Avg. Hollingshead score = 44.7 (SD = 10.6)	*Individual Factors*: • Children with ASD had improvements in sleep latency, sleep efficiency, and overall child QoL post sleep education • Children with ASD had improvements in repetitive behavior post sleep education *Family Factors*: • Parent sleep education for children with ASD was associated with improved parenting sense of competence
25– Martin et al. (2021)	*N* = 234; Mean Age = 8.79 *±* 2.11 yrs., Range 5–13 yrs.; 64.5% Male (*n* = 151); Race/Ethnicity NR; Australia	*Caregiver Highest Education*: 64.1% completed tertiary study (*n* = 150), 24.8% HS only (*n* = 58), 11.1% did not complete HS (*n* = 26); *Household Structure*: 24.4% Single parent home (*n* = 57); *SEIFA Australia*: 1033.03 (SD = 55.74, range: 795–1117)	*Family Factors*: • Associations were found between sleep initiation and duration and maternal mental health • Specific child sleep problems were not associated with HRQoL or parenting stress *Neighborhood and Socio-cultural Factors*: • Child sleep problems and an area deprivation index, reflecting neighborhood-level SES, were associated with poorer maternal well-being, but associations between this index and sleep problems were not assessed
26– Masi et al. (2022)	*N* = 969; Age Range = 2–17 yrs.; 78.7% Male (*n* = 763); Race/Ethnicity NR; Australia	*Family Income (Annual)*: 29.8% <$70k (*n* = 234), 25.6% $70k to $104k (*n* = 203), 44.3% >$104k (*n* = 348)	*Individual Factors*: • Children with ASD have a greater severity of sleep problems compared to non-ASD siblings and unrelated children • Females had greater bedtime resistance, reduced sleep duration, increased sleep anxiety, and daytime sleepiness than males *Family Factors*: • Low family income was linked to greater severity of sleep problems
27– Mazurek and Petroski (2015)	*N* = 1,347; *2–5 yrs. (n* *= 461)*: Mean Age = 4.69 *±* 0.79 yrs.; 82.9% Male (*n* = 382); 90.2% White (*n* = 403); 9.8% Other Race (*n* = 44); *6–18 yrs. (n* *= 886)*: Mean Age = 9.60 *±* 2.91 yrs.; 85.9% Male (*n* = 761); 90.8% White (*n* = 779); 9.2% Other Race (*n* = 79); US & Canada	*Caregiver Highest Education (Age 2–5 yrs.)*: 0.7% some HS (*n* = 3), 7.9% HS (*n* = 34), 28.3% some college (*n* = 122), 30.9% Bachelor’s degree (*n* = 133), 32.2% Postgraduate education (*n* = 139); *Caregiver Highest Education (Age 6–18 yrs.)*: 1.1% some HS (*n* = 9), 9.4% HS (*n* = 80), 30.4% some college (*n* = 259), 30.4% Bachelor’s degree (*n* = 259), 28.8% Postgraduate education (*n* = 246)	*Individual Factors*: • Children with anxiety and sensory over-responsivity are at risk for sleep problems • Hyperarousal may be an underlying mechanism of sleep disturbance for some children with ASD • Anxiety was associated with bedtime resistance, sleep-onset delay, sleep duration, sleep anxiety, and night wakings
28– McLay et al. (2020)	*N* = 244; Age NR; Sex NR; 77% New Zealand European/Pakeha (*n* = 187), 6.6% Maori (*n* = 16), 4.8% Asian (*n* = 11), 10.7% Other (*n* = 26); New Zealand, US, & Australia	*Caregiver Highest Education*: 66% University degree (*n* = 160); *Caregiver Employment*: 52% Employed (*n* = 127), 25% Homemaker (*n* = 60), 12.3% Self-employed (*n* = 30), 6.1% Unable to work (*n* = 15), 3.7% Student (*n* = 9), 1.2% Retired (*n* = 3); *Household Structure*: 78.3% married (*n* = 191), 12% divorced/separated (*n* = 29), 7% single (*n* = 17), 0.8% same sex (*n* = 2), 1.2% widowed (*n* = 3); *Family Income (Annual)*: 73% $50 to $100k+ (*n* = 179); *Family Size*: 6.6% one (*n* = 16), 20.5% two (*n* = 50), 43% three (*n* = 105), 30% 4 + members (*n* = 72)	*Individual Factors*: • Parents believed that child’s sleep problems were linked to intrinsic ASD features and were part of who they (children) were and were unlikely to change without treatment *Family Factors*: • Parental attributions about child sleep: 67% of parents reported they tried a sleep intervention for their child more than once and parents reported trying on average six different treatments for sleep problems and 68% tried medication to treat sleep problems
**29–** Papadopoulos et al. (2021)	*N* = 56; *ASD + ID (n* *= 34*,* 91%)*: Mean Age = 8.91 *±* 2.21 yrs.; 91.2% Male (*n* = 31), Race and Ethnicity NR; *ASD (n* *= 22*,* 73%)*: Mean Age = 9.59 *±* 2.26 yrs.; 72.7% Male (*n* = 16), Race and Ethnicity NR; Australia	*ASD + ID*: *Caregiver Highest Education*: 8.8% Completed year 10 (*n* = 3), 8.8% completed HS (*n* = 3), 41.2% completed tertiary school (*n* = 14); *Marital Status*: 79.4% couple (*n* = 27), 20.6% Single (*n* = 7); *Caregiver Employment*: 67.6% employed (*n* = 23); *Household Structure*: 76.5% lives with both parents (*n* = 26); *ASD*: *Caregiver Highest Education*: 9.1% Completed year 10 (*n* = 2), 50% completed tertiary school (*n* = 11); *Marital Status*: 86.4% couple (*n* = 19), 13.6% single (*n* = 3); *Caregiver Employment*: 54.5% employed (*n* = 12); *Household Structure*: 86.4% lives with both parents (*n* = 19)	*Individual Factors*: • Emotional and behavioral problems were associated with sleep problems in children with ASD and ASD + ID • In children with ASD + ID and ASD, emotional and behavioral problems were associated with sleep problems *Family Factors*: • In children with ASD, parent mental health was associated with sleep problems
**30–** Pattison et al. (2022)	*N* = 123; Mean Age = 8 *±* NR yrs., Range = 5–13 yrs.; 65% Male; Race/Ethnicity NR; Australia	*Caregiver Highest Education*: 67% completed tertiary study (*n* = 82)	*Individual Factors*: • Barriers to implementation of the sleep intervention included child illness and anxiety *Family Factors*: • Barriers to implementation of the sleep intervention included parent anxiety • Facilitators to implementation of the sleep intervention included family support and consistency and patience in implementing strategies • Parents valued practical child sleep resources tailored to the family
31– Phung et al. (2019)	*N* = 20–28; *Study 1 (n* *= 28)*^*a*^: Mean age = 14.64 *±* 1.97 yrs., Range = 12–18 yrs.; 89.3% Male; 45.2% White, 35.5% Multiracial/other, 19.3% Hispanic/Latino; *Study 2 (n* *= 20)*^*a*^: Mean age = 16.74 *±* 2.52 yrs., range 11–20 yrs.; 80% Male; 45% White, 30% Multiracial/other, 25% Hispanic/Latino; US	NR	*Individual Factors*: • Sleep quality and amount were associated with depressive symptoms *Family Factors*: • Daytime sleepiness was correlated with mother-adolescent discordance and depressive symptoms • Adolescent-reported discordance with siblings was correlated with objective sleep measures
32– Richdale and Schreck (2009)	—	—	*Individual Factors*: • Some children with ASD have a disturbance in melatonin production and circadian timing • Insomnia in children with ASD is linked to unfavorable bedtime routines and disruptive behaviors *Family Factors*: • Sleep problems in children with ASD can increase psychological distress of family members
33– Roberts et al. (2017)	*N* = 70; Mean Age = 7.32 *±* NR yrs., Range 4–12 yrs.; 75.7% Male (*n* = 53); 80% White (*n* = 56); US	*Family’s Financial Status*: 8.57% Unable to meet financial needs (*n* = 6), 38.57% Can consistently meet financial needs (*n* = 27); *Household Structure*: 18.57% Single-parent household (*n* = 13)	*Individual Factors*: • Children with ASD + ADHD had worse sleep than those without ADHD • Child age, time since ASD diagnosis, and medication use was not associated with sleep *Family Factors*: • Family strain, family distress, and negative resilience were higher in families of children with sleep problems
34– Shui et al. (2023)	*N* = 950; *All cohorts*: Age Range = 2–5 yrs.; 80.6% Male (*n* = 766), 82.8% White (*n* = 764), 17.2% Non-White (*n* = 159); US & Canada	*Caregiver Highest Education*: *Completed at least some college*: Cohort 1: 86% (*n* = 86), Cohort 2: 83% (*n* = 166), Cohort 3: 84.8% (*n* = 156), Cohort 4: 81.5% (*n* = 163), Cohort 5: 81.1% (*n* = 198); *Completed at most HS*: Cohort 1: 14% (*n* = 14), Cohort 2: 17% (*n* = 34), Cohort 3: 15.2% (*n* = 28), Cohort 4: 18.5% (*n* = 37), Cohort 5: 18.9% (*n* = 46)	*Individual Factors*: • Significant factors in developing sleep problems are self-injurious behavior, sensory issues, and dental problems • Predictors of future sleep problems include dental problems, racial/ethnic status, sensory issues, and self-injurious behavior • Racial and ethnic minoritized background was associated with poorer child sleep *Family Factors*: • A significant factor in developing sleep problems was lower caregiver education longitudinally
35– Singer et al. (2019)	Commentary based on Tomkies et al. (2019)	—	*Individual Factors*: • Children with OSA did not have differences in demographics or clinical characteristics compared to those without OSA • Hispanic ethnicity and African American race were associated with severe OSA, but more likely due to higher weight rather than ASD
36– Tomkies et al. (2019)	*N* = 45; Mean Age = 6.1 *±* 2.8 yrs., range 2–14 yrs.; 80% Male (*n* = 36); 49% Hispanic/Latino (*n* = 22), 27% Black or African American (*n* = 12), 22% White (*n* = 10), and 2.2% other (*n* = 1); US	NR	*Individual Factors*: • 58% of the children had OSA and of those 35% had severe OSA • Comorbidities like seizure, cerebral palsy, and ADHD were not determined to be predictors of OSA • Higher weight was a predictor of severe OSA and Hispanic and African American children were more likely to be obese
37– Waddington et al. (2020)	*N* = 203; Mean age = 8.47 *±* 4.08 yrs., range 2–18 yrs.; 80% Male (*n* = 163); Race and Ethnicity NR; Australia	*Family Income*: 25% <$70k (*n* = 49), 26% $70k to $104k (*n* = 51), 41% >$104k (*n* = 82), 9% Prefer not to say (*n* = 17); *Maternal Highest Education*: 16% < 12 yrs. (*n* = 32), 22% 12 yrs. (*n* = 45), 21% Trade/technical certificate (*n* = 43), 41% Completed/completing university (*n* = 83); *Paternal Highest Education*: 21% < 12 yrs. (*n* = 41), 13% 12 yrs. *(n* = 26), 32% Trade/technical certificate (*n* = 63), 33% Completed/completing university (*n* = 65)	*Individual Factors*: • Increased sleep disturbance was associated with greater ASD symptom severity, anxiety, depression, and child seizures *Family Factors*: • Increased sleep disturbance was associated with lower paternal education, lower family income, and maternal ASD traits • Strongest predictor of sleep disturbance was maternal ASD traits
38– Wang et al. (2016)	*N* = 60; Mean age = 11.53 *±* 2.92 yrs., range 6–17 yrs.; 83.3% Male (*n* = 50); Race and Ethnicity NR; China	*Caregiver Highest Education*: 53.7% Undergraduate degree or above (*n* = 29), 46.3% HS or below (*n* = 25); *Family Income*: 62% < 100,000 RMB Yuan (*n* = 31), 38% ≥ 100,000 RMB Yuan (*n* = 19); *Household Structure*: 86.5% Two parents (*n* = 45), 9.6% Single parent (*n* = 5), 3.8% Remarried or Other (*n* = 2)	*Individual Factors*: • Sleep disturbance was correlated with poorer prosocial behavior, higher hyperactivity, and female sex *Family Factors*: • Sleep disturbance was correlated with older parental age • Parental education, marital status, family income, living space, noise in the house, and co-sleeping *did not* predict child sleep disturbance
39– Whelan et al. (2022)	—	—	*Individual Factors*: • Children with ASD with sleep problems had more disruptive behavior, such as irritability, hyperactivity, and aggression • Poor sleep was linked to anxiety and emotional problems • Sleep duration and sleep latency were linked to social functioning • ASD symptom severity was increased by short sleep duration
40– Williams et al. (2004)	*N* = 210; Mean Age = 8.4 *±* NR yrs., range 2–16 yrs.; 80.5% Male (*n* = 169); Race and Ethnicity NR; 63% were designated “MR” (*n* = 127); US	NR	*Individual Factors*: • Difficulty falling asleep, restless sleep, not falling asleep in own bed, and frequent night wakings were the most frequently reported sleep problems • Nighttime wakings were significantly more common in the “MR group” versus the “not MR group” • No difference in sleep problems related to the child’s age except for nocturnal enuresis • Vision problems, runny nose, and upper respiratory problems were associated with decreased sleep • Vision problems, poor appetite, and poor growth were associated with increased night wakings • Decreased willingness to fall asleep was associated with poor appetite and poor growth
41– Won et al. (2019)	*N* = 1,069; Mean age = 7.7 *±* 3.8 yrs.; 81% Male (*n* = 856); 66.9% White (*n* = 651), 20.5% Black (*n* = 199), 5.0% Asian or Pacific Islander (*n* = 49), 7.6% Other Race (*n* = 74); 79.3% non-Hispanic/Latino (*n* = 772); US	*Caregiver Highest Education*: 45.4% College graduate and above (*n* = 316), 44.3% HS graduate/some college (*n* = 308), 10.3% < HS (*n* = 72); *Medical Insurance*: 49.9% private (*n* = 487), 45.0% public (*n* = 439), 2.3% public + private (*n* = 22), 2.9% other (*n* = 28)	*Individual Factors*: • Sleep problem documentation differed by age group • Maladaptive behaviors may take precedence over sleep problems in children with ASD + ADHD, which results in under-reporting of sleep problems by pediatricians *Neighborhood and Socio-cultural Factors*: • Sleep problems differed by medical insurance type and site • Public + private medical insurance was associated with more sleep problems

Note. Categories and *n* (%) of race, ethnicity, and SES indicators displayed as reported in the study for children with ASD. For readability, categories with 0% were not listed and age was reported in years. ^*a*^ Sample size included children with ASD. Bolded studies included an intervention. NR = not reported. ADHD, AD/HD = attention-deficit/hyperactivity disorder; ASD = autism spectrum disorder; GCSE = General Certificate of Secondary Education; HS = high school; HRQoL = health-related quality of life; ID = intellectual disability; OSA = obstructive sleep apnea; QoL = quality of life; MR = “mentally retarded”; SEIFA = Socio-economic index for area; SES = socioeconomic status; TD = typically developing

## Study Design Characteristics

The sample size of children with ASD ranged from 1 (Davenport et al., 2021) to 5,787 (Ezell et al., 2016). The mean number of participants was 693 (*SD* = 1,357). The majority of articles excluding reviews and commentaries (*n* = 6), had fewer than 200 (*n* = 23, 65.7%) participants, and a few studies included more than 500 participants (*n* = 8, 22.9%). Of 41 articles included, 18 (43.9%) were descriptive/cross-sectional, 4 (9.8%) longitudinal, 7 (17.1%) retrospectives, 3 (7.3%) RCTs, 2 (4.9%) pilot feasibility, 2 (4.9%) literature reviews, 2 (4.9%) systematic reviews, 1 (2.4%) case study, 1 commentary (2.4%), and 1 (2.4%) meta-analysis; 6 (14.6%) included an intervention to promote sleep health in children with ASD. Of the included studies, 26 (63.4%) included children with ASD only; 1 (2.4%) included children with ASD + ID (Papadopoulos et al., 2021); 9 (22.0%) included children with ASD and a comparison group; 3 (7.3%) included children with ASD and children with ASD + ADHD (Leader et al., 2022a; Roberts et al., 2017; Won et al., 2019); and 1 (2.4%) included children with ASD with or without ADHD or ID (Whelan et al., 2022).

## Sociodemographic Information and Inclusion of Other Health Disparity Groups

The sociodemographic information is reported in Table 1. Included children had a mean age of 8.0 years (SD = 2.62), Range = 1 to 20 years; 78.4% were male (*n* = 34). The majority of studies included school-age children (6 to 12 years; 26/41, 63.4%). Of the articles included, excluding reviews and commentaries, 17 (48.6%) studies were based in the US, 14 (40%) were conducted outside of the US, 3 (8.6%) were based in the US and Canada, and 1 (2.9%) was based in the US, New Zealand and Australia.

Representation of NIMHD-defined racial and ethnic minoritized groups was limited across studies. Overall, excluding reviews and commentaries, only about half (*n* = 17, 48.6%) of studies reported race and ethnicity data. On average across the 17 studies reporting race and ethnicity data, the majority (66%) of participants identified as non-Hispanic/Latinx white, with a range of 0% (Davenport et al., 2021) to 90.8% (Mazurek & Petroski, 2015).

Most (*n* = 27, 77.1%) studies reported at least one indicator of SES. Of these 27 studies, caregiver’s highest level of educational attainment was most often reported (*n* = 21, 77.8%), with most samples consisting of caregivers with higher levels of educational attainment (i.e., at least some college or beyond). Nearly half of studies (*n* = 13, 48.1%) reported on at least 2 indicators of SES. Household structure, including caregivers’ marital status, was reported in 11 studies (40.7%), with 6 studies reporting caregiver employment status (22.2%) and 6 reporting family income (22.2%). Of studies reporting income-related SES indicators, most participants reported higher levels of family income, with some exceptions (e.g., Galli et al., 2022). A few (*n* = 3) studies used established indices of SES, such as the Hollingshead index, while 2 studies conducted in Australia reported indexes reflecting neighborhood-level SES (Baker et al., 2023; Martin et al., 2021). Two US-based studies reported insurance status, with 55.1% of one sample publicly insured (Broder-Fingert et al., 2014) and 45.0% publicly insured in another sample (Won et al., 2019). Only 1 study (Johnson et al., 2023) reported on the proportion of participants living in rural settings, which represented nearly two-thirds of that study’s sample and is another designated health disparity population.

## ASD and Sleep Constructs Included

Measures in the extracted studies predominately included diagnostic/screening assessments for ASD and parent ratings of child sleep.

### Autism Diagnostic Screening and Assessment Measures

Of the 41 included studies, 16 (45.7%) used a clinical diagnosis (e.g., DSM-V, DSM-IV) and/or screening measures for ASD, 8 (22.9%) used the Autism Diagnostic Observation Schedule, 4 (11.4%) used parent-report, 3 (8.6%) used the medical record, 2 (5.7%) used the Gilliam Autism Rating Scale, 1 (2.9%) used the Child Autism Rating Scale, and 1 (2.9%) used the Autism Diagnostic Interview-Revised.

### Child Sleep

The most commonly used measure of child sleep (*n* = 19, 54.3%) was the Children’s Sleep Habits Questionnaire (CSHQ; Owens et al., 2000). Six (17.1%) studies measured child sleep with actigraphy, 11 (31.4%) used parent ratings on a general list of questions, 7 (20%) used a sleep diary, 2 (5.7%) used the Family Inventory of Sleep Habits (FISH; Malow et al., 2009), 1 (2.9%) used the Sleep Habits Survey (SHS; Wolfson & Carskadon, 1998), 1 (2.9%) used the Clinician Global Impression-Severity (CGI-S; Guy, 1976), 1 (2.9%) used the Modified Simonds and Parraga Sleep Questionnaire (MSPSQ; Simonds & Parraga, 1982), 1 (2.9%) used the BEARS (Bedtime, Excessive Daytime Sleepiness, Awakening During the Night, Regularity and Duration of Sleep, and Snoring; Owens & Dalzell, 2005), and 1 (2.9%) used the Sleep Disturbance Scale for Children (SDSC; Bruni et al., 1996).

### Socio-Ecological Factors Linked To SHDs in Children with ASD

**Fig. 2:**
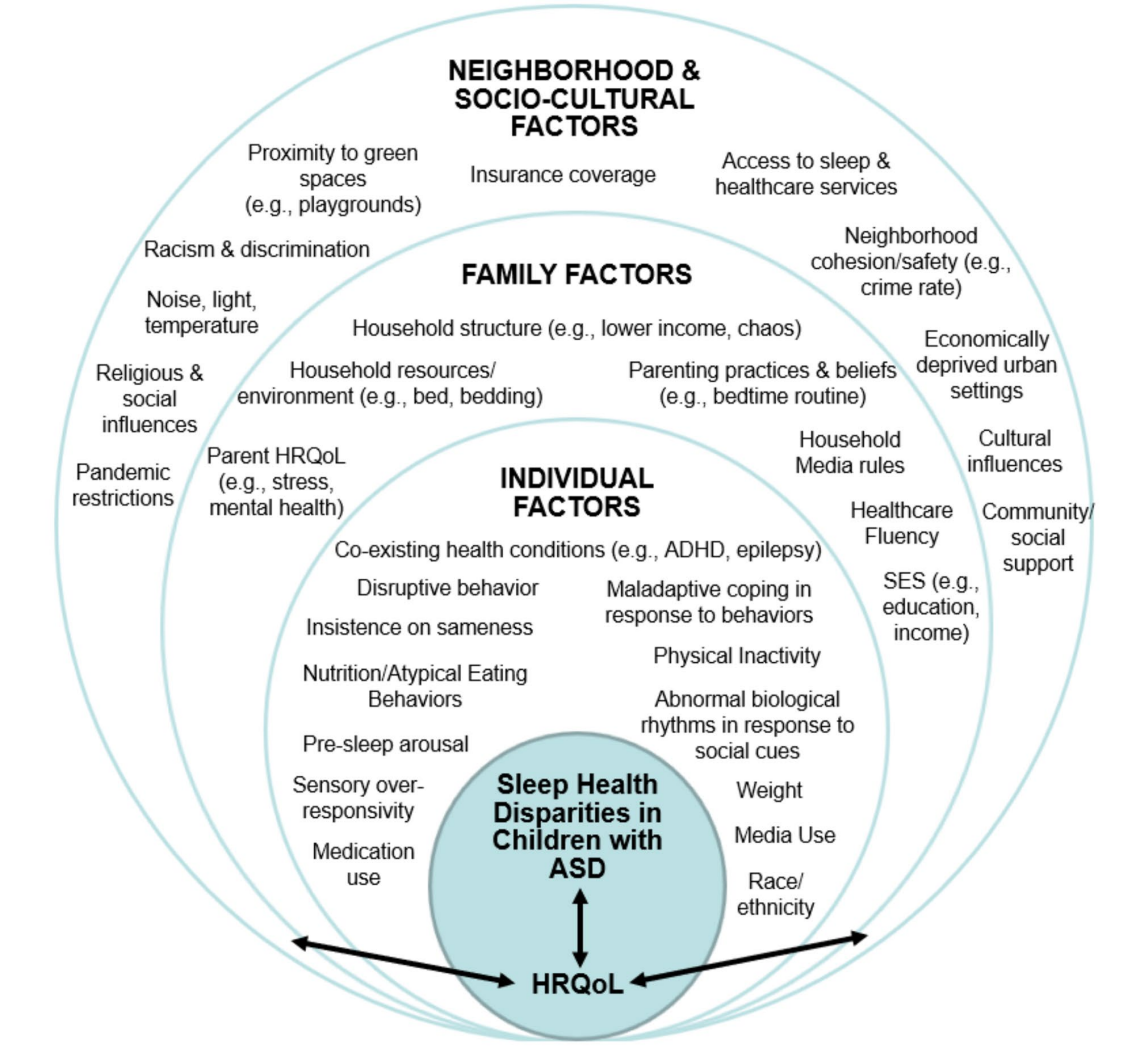
Multi-level socio-ecological factors contributing to sleep health disparities in children with ASD (Bronfenbrenner & Ceci, 1994; Golden & Earp, 2012). Adapted with permission from Billings et al. (2021) (License Number: 5817710303222; Date: 6/28/24). ADHD = attention-deficit/hyperactivity disorder; HRQoL = health-related quality of life; SES = socioeconomic status

Of the extracted articles (see Table 1), 31 (75.6%) focused on individual factors, 27 (65.9%) focused on family factors, and 11 (26.8%) focused on neighborhood and broader socio-cultural factors. Only 3 (7.3%) studies included factors at all three socio-ecological levels (Durán-Pacheco et al., 2023; Johnson et al., 2023; MacDonald et al., 2021). Figure 2 includes an overview of the possible multi-level socio-ecological factors contributing to SHDs in ASD.

### Individual Factors

*Race/ethnicity*. Shui et al. (2023) examined child race and ethnicity in relation to sleep problems, finding that those of racial and ethnic minoritized backgrounds were more likely to experience poor sleep. However, the sample was primarily non-Latinx White, and analysis was limited to comparing “White” versus “non-White” children. Tomkies et al. (2019) examined the relationship between race and ethnicity and obstructive sleep apnea in children with ASD, but did not find any differences. However, Tomkies et al. (2019) did find that higher weight was a predictor of severe obstructive sleep apnea, and Hispanic and African American children were more likely to be obese. *ASD symptomatology.* Han et al. (2022) and Ezell et al. (2016) identified links between sleep problems and executive functioning, adaptive functioning, attention problems, and core ASD symptoms. *Behavioral problems*,* emotions*,* and ADHD.* Galli et al. (2022) identified an association between sleep disorders and emotional and behavioral problems. Leader et al. (2022) found that children with ASD and ADHD had more bedtime settling difficulties than children with ASD without ADHD. *Physical activity.* Elkhatib Smidt et al. (2022) and Garcia et al. (2020) identified links between greater physical activity and longer sleep duration in ASD. Garcia et al. (2020) measured physical activity using wrist actigraphy, while Elkhatib Smidt et al. (2022) used a single caregiver-report item.

### Family Factors

*Family SES Indicators*. Durán-Pacheco et al. (2023), Masi et al. (2022), and Waddington et al. (2020) found that lower family/household income was associated with greater sleep problems in children with ASD. Roberts et al. (2017) also found that perceived family financial strain was higher among families of children with sleep problems. Lower levels of caregiver educational attainment were also associated with greater sleep problems in three studies (Elkhatib Smidt et al., 2020; Shui et al., 2023; Waddington et al., 2020). However, Wang et al. (2016) found that neither family income nor caregiver educational attainment was linked to sleep disturbance. *Maternal/parental mental health and HRQoL.* Roberts et al. (2017) showed that families of children with sleep problems had higher strains, distress, and reduced resilience compared to children without sleep problems. Durán-Pacheco et al. (2023) found that the severity of ASD affects both the child and caregiver HRQoL and identified a link between increased ASD severity and reduced child sleep quality.

### Neighborhood and Broader Socio-Cultural Factors

*Cross-cultural differences*. A study conducted by Jeon et al. (2023) in South Korea and the United Kingdom found that practices influenced by culture, like co-sleeping, may influence bedtime and sleep duration in children with ASD. Jeon et al. (2023) also reported that parents of South Korean students with ASD reported later bedtimes and decreased sleep duration due to increased academic demands. *COVID-19 pandemic.* Sleep patterns were disrupted in children with ASD due to increased media use during the pandemic (Bruni et al., 2022). Lewis et al. (2023) additionally found that sleep problems worsened for children with ASD during the pandemic including: reduced overall sleep duration and quality, difficulty falling asleep, frequent night awakenings, and difficulty waking. *Insurance.* Children with publicly funded insurance and a sleep disorder had a higher odds of obesity compared to children with privately funded insurance (Broder-Fingert et al., 2014), but associations between insurance coverage and sleep disorders were not examined. Won et al. (2019) additionally found evidence of increased sleep problem documentation among children with both public and private medical insurance coverage. *Neighborhood-level SES.* Martin et al. (2021) found that both sleep problems and an area deprivation index, reflecting neighborhood-level SES, were associated with poorer maternal well-being, but associations between this index and sleep problems were not assessed.

## Possible Gaps/Limitations in Existing Literature on SHDs in Children with ASD

Several strengths were mentioned across studies including large population-based longitudinal studies (Baker et al., 2023; Shui et al., 2023; Won et al., 2019), large cross-sectional studies (Elkhatib Smidt et al., 2022; Mazurek & Petroski, 2015; McLay et al., 2020; Waddington et al., 2020; Williams et al., 2004), and a cross-cultural comparative analysis (Bin Eid et al., 2022). Several limitations were also mentioned including the use of a parent-reported (Elkhatib Smidt et al., 2022) or medical record diagnosis of ASD (Baker et al., 2023; Broder-Fingert et al., 2014; Martin et al., 2021; McLay et al., 2020; Papadopoulos et al., 2021; Roberts et al., 2017; Tomkies et al., 2019; Wang et al., 2016), lack of variability in demographics (McLay et al., 2020), and subjective report of lifestyle factors, specifically meal time regularity (Bin Eid et al., 2022).

## Recommendations for Future Interventions and Possible Solutions to Address SHDs in Children with ASD

Six (14.6%) studies included a sleep intervention for children with ASD (Davenport et al., 2021; Johnson et al., 2023; MacDonald et al., 2021; Malow et al., 2014; Papadopoulos et al., 2022; Pattison et al., 2022). *Individual factors.* All studies showed improvements in some aspects of sleep disturbance in children with ASD post sleep intervention. Pattison et al. (2022) found that children who received the sleep intervention reported greater reduction in bedtime resistance, sleep onset delay, sleep duration, and parasomnias compared to controls. MacDonald et al. (2021), Malow et al. (2014), Davenport et al. (2021), and Papadopoulos et al. (2022), found improvement in aspects of daytime behavior in children with ASD post sleep intervention. However, Johnson et al. (2023) did not find improvements in irritability post sleep intervention. At 12-months follow up, Pattison et al. (2022) also did not find improvements in child behavioral functioning post sleep intervention. *Family factors.* Johnson et al. (2023) and Malow et al. (2014) found that parents had improvements in their sense of competency post sleep intervention. *Broader neighborhood and socio-cultural factors.* Davenport et al. (2021) and Johnson et al. (2023) showed that telehealth-delivered sleep treatments are promising for improving access to care for children with ASD and their families who may reside in rural and medically underserved areas.

## Discussion

Using a socio-ecological framework, this scoping review examined factors linked to SHDs in children with ASD, as well as the representation of other health disparity groups in this research, and recommendations for future clinical research. Most studies focused on individual child factors associated with sleep problems in children with ASD, with less research focused on family factors, and very few studies examining broader neighborhood and socio-cultural factors. In addition, only about half of studies reported race and ethnicity data, with sparse representation of NIMHD-defined racial and ethnic minoritized children and families overall. Although many studies included indicators of SES, these were not consistently examined in relation to child sleep, and most samples were primarily of higher-SES backgrounds and/or educational attainment. Collectively, these findings highlight the need for future research on modifiable socio-ecological factors to guide equitable sleep interventions for children with ASD, and particularly those with overlapping marginalized identities (e.g., ASD as well as racial and ethnic minoritized and/or lower-SES backgrounds).

Individual child factors (e.g., epilepsy, anxiety) were linked to greater sleep problems and could result in delayed access to sleep interventions (Herrmann, 2016; Leader et al., 2022a; Levin & Scher, 2016; Mazurek & Petroski, 2015; Tye et al., 2018). For example, parents of children with severe disruptive behaviors may prioritize behavioral therapy prior to seeking treatment for sleep (Chen et al., 2021). In contrast, delayed access to early intervention for disruptive behavior may result in increased arousal and dysregulation contributing to disruptions in the child’s sleep-wake cycle (Masi et al., 2022).

Regarding family-level socio-ecological factors, family structure, and knowledge about sleep were associated with sleep problems in children with ASD. Studies showed that raising a child with ASD was associated with higher levels of parenting stress and increased child sleep problems (Herrmann, 2016; Levin & Scher, 2016). Children’s sleep habits also predicted worse maternal mental health outcomes, poor maternal sleep quality, and increased maternal stress (Hodge et al., 2013). Given these findings, future studies should consider examining family perspectives and understanding of child sleep to inform family-centered and culturally-responsive sleep interventions for children with ASD and their caregivers.

In addition, family-level indicators of lower SES, including lower household income, perceived financial strain, and lower levels of caregiver educational attainment, were generally associated with greater sleep problems (Durán-Pacheco et al., 2023; Elkhatib Smidt et al., 2020; Masi et al., 2022; Roberts et al., 2017; Shui et al., 2023; Waddington et al., 2020), with the exception of one study (Wang et al., 2016). Wang et al. (2016) found that neither family income nor caregiver educational attainment was linked to sleep disturbances. Two possible explanations for the conflicting results reported by Wang et al. (2016) in their study conducted in China is that over half of the sample of parents in the sample were college graduates or higher, and most of the children lived with two married parents. These findings are consistent with research on socioeconomic SHDs in general pediatric populations (Covington et al., 2021; Newton et al., 2020), although future research is needed to identify whether children with ASD and lower-SES backgrounds exhibit worse sleep than neurotypical children with lower-SES backgrounds. In addition, neighborhood-level SES and healthcare-related factors, such as insurance coverage, were rarely examined and should be included in future work.

Several strengths were evident across studies including large population-based longitudinal studies and large cross-sectional study designs. However, gaps and potential limitations emerged in this review, as most studies were descriptive/cross-sectional. In addition, only three studies examined factors across all three levels of the socio-ecological framework (Durán-Pacheco et al., 2023; Johnson et al., 2023; MacDonald et al., 2021). Future studies should consider measuring and evaluating interactions between individual, family, and neighborhood and socio-cultural factors in making recommendations for sleep health promotion and support in children with ASD.

The third and final aim of this review was to suggest recommendations for interventions and identify possible solutions to mitigate SHDs in children with ASD, as these are important next steps after identifying potential socio-ecological factors contributing to SHDs in ASD. In this review, studies found that improved sleep was associated with decreased daytime disruptive behavior and improvements in bedtime and sleep disturbance (Davenport et al., 2021; Johnson et al., 2023; MacDonald et al., 2021; Malow et al., 2014). Davenport et al. (2021) and Johnson et al. (2023) demonstrated that telehealth-delivered sleep treatments show promise in improving access to care for children with ASD and their families in rural and medically underserved areas, which provides an excellent basis for future research. However, these studies had a small number of racially or ethnically diverse families. To our knowledge, no sleep intervention studies have specifically focused on children with ASD and their families who experience racism and discrimination due to having a racial and ethnic minoritized and/or lower-SES background. Additional research in this regard may inform more equitable personalization of sleep interventions, in line with calls for more evidence-based and person-centered sleep treatment for children with ASD (Papadopoulos et al., 2022). Studies evaluating the extent of culturally-responsive and equitable sleep interventions for youth with ASD are also needed.

## Conclusions

Given the high prevalence of sleep problems in children with ASD and multi-level socio-ecological factors linked to SHDs, interdisciplinary efforts are needed to equitably prevent, identify, and treat sleep problems in children with ASD and their families. Although additional systemic changes (i.e., policy, neighborhood, healthcare-related) are needed to promote sleep health equity more broadly (Jackson et al., 2020), future research should focus on developing and testing person- and family-centered interventions tailored to address modifiable factors contributing to SHDs in children with ASD.

## Electronic supplementary material

Below is the link to the electronic supplementary material.

Supplementary Material 1
