# Supplementary material for: Scoping Review of Socio-Ecological Factors Contributing to Sleep Health Disparities in Children with Autism Spectrum Disorder
Source: J Autism Dev Disord. 2025 Apr 5;55(12):4390–411. doi: 10.1007/s10803-025-06807-x (PMC12589370; doi:10.1007/s10803-025-06807-x)
Supplement: Supplementary file 1 — Supplementary Material 1 [file 10803_2025_6807_MOESM1_ESM.docx]

**Appendix A.** *Comprehensive List of Keywords and Search Strategy*

**Database(s): Ovid MEDLINE(R) and Epub Ahead of Print, In-Process, In-Data-Review & Other Non-Indexed Citations and Daily 1946 to September 28, 2023**

# Searches Results

1 autism spectrum disorder/ or asperger syndrome/ or autistic disorder/ 42979

2 (autism or autistic*or asperger*).ti,ab. 60373

3 or/1-2 67614

4 exp sleep wake disorders/ or dyssomnias/ or sleep disorders, circadian rhythm/ or sleep disorders, intrinsic/ or "disorders of excessive somnolence"/ or idiopathic hypersomnia/ or restless legs syndrome/ or sleep apnea syndromes/ or sleep/ or sleep deprivation/ or sleep duration/ or sleep hygiene/ or sleep quality/ or sleep latency/ or sleep stages/ 164903

5 exp Sleep Medicine Specialty/ 407

6 exp Narcolepsy/ 4389

7 exp Nocturnal Myoclonus Syndrome/ 677

8 sleep*.ti,ab. 233510

9 night awaking.ti,ab. 4

10 night waking.ti,ab. 347

11 waking at night.ti,ab. 65

12 night terror*.ti,ab. 260

13 bedtime.ti,ab. 6532

14 (insomnia or dyssomnia* or parasomnia* or hypersomnia or hypersomnolence or somnolence or apnea or circadian or wakefulness).ti,ab. 142355

15 (narcolepsy or narcoleptic*).ti,ab. 5494

16 (nocturnal adj2 myoclonus).ti,ab. 109

17 "Periodic Limb Movement".ti,ab. 542

18 (Willis adj2 Ekbom).ti,ab. 177

19 (Wittmaack adj2 Ekbom).ti,ab. 2

20 (restless adj2 leg*).ti,ab. 5587

21 tired*.ti,ab. 7188

22 or/4-21 341793

23 health inequities/ or health status disparities/ or socioeconomic disparities in health/ or exp Healthcare Disparities/ 39385

24 exp ethnicity/ or exp racial groups/ or exp "health disparate, minority and vulnerable populations"/ or "black or african american"/ or amish/ or arabs/ or "asian american native hawaiian and pacific islander"/ or asian/ or "native hawaiian or other pacific islander"/ or "hispanic or latino"/ or mexican americans/ or indigenous peoples/ or jews/ or "sexual and gender minorities"/ or transgender persons/ or vulnerable populations/ 252636

25 culture/ or cultural diversity/ or diversity, equity, inclusion/ or exp Cultural Characteristics/ 62371

26 exp social environment/ or exp home environment/ or exp neighborhood characteristics/ or exp social marginalization/ or exp social vulnerability/ or exp sociodemographic factors/ 128991

27 rural population/ or suburban population/ or urban population/ 114401

28 exp socioeconomic factors/ or economic factors/ or economic status/ or exp poverty/ or poverty areas/ or exp social class/ 513821

29 Health Services Accessibility/ or exp Insurance/ or Medically Uninsured/ 276369

30 exp Food Deserts/ 43

31 exp Educational Status/ 61814

32 Income/ 34902

33 exp Systemic Racism/ or exp Racism/ 6642

34 exp Single-Parent Family/ 580

35 (inequit* or disparit* or socioeconomic*).ti,ab. 223436

36 at risk population*.ti,ab. 6183

37 (ethnic* or Mexican American* or Hispanic or Latin*).ti,ab. 262207

38 (racial group* or Black* or African American* or Asian* or Native American* or Indigenous or Amish or Native Hawaiian or Arab* or Jews or Jewish or lgbtq or trangender* or gender minorit* or vulnerable population*).ti,ab. 587449

39 (cultur* or diversity or equity or inclusion).ti,ab. 1925481

40 (neighborhood* or housing or unhoused or homeless* or marginaliz* or social vulnerabilit* or sociodemographic* or rural or suburban or urban or city or cities or segregation or redlining or playground* or connectedness).ti,ab. 670560

41 (green adj2 space*).ti,ab. 2555

42 (recreation adj2 center*).ti,ab. 181

43 (home adj2 environment).ti,ab. 7155

44 (social adj2 environment).ti,ab. 9993

45 (socioeconomic* or inequalit* or economic* or poverty or social class*).ti,ab. 536970

46 (insurance or uninsured or medicaid).ti,ab. 139958

47 Health Services Accessibility.ti,ab. 64

48 (care adj2 barrier*).ti,ab. 6015

49 access to care.ti,ab. 16101

50 access to resources.ti,ab. 2435

51 (food desert* or food apartheid or food insecurity).ti,ab. 8017

52 (educational adj2 status).ti,ab. 6841

53 (educational adj2 level).ti,ab. 22934

54 (parental adj2 education).ti,ab. 5332

55 income.ti,ab. 176397

56 racism.ti,ab. 8103

57 single parent*.ti,ab. 3084

58 single father*.ti,ab. 116

59 single mother*.ti,ab. 1397

60 or/23-59 4347923

61 adolescent/ or child/ or child, preschool/ 3379756

62 exp Pediatrics/ 63144

63 (adolescen* or adolescent* child* or preschool* or boy* or girl* or teen* or youth* or juvenile* or pediatric* or paediatric*).ti,ab. 1141347

64 or/61-63 3746859

65 3 and 22 and 60 and 64 195

**APA PsycInfo 1806 to September Week 3 2023**

# Searches Results

1 exp Autism Spectrum Disorders/ 55688

2 (autism or autistic*or asperger*).ti,ab. 57861

3 or/1-2 65136

4 exp sleep wake disorders/ or hypersomnia/ or insomnia/ or narcolepsy/ or parasomnias/ or sleep apnea/ 24427

5 exp restless leg syndrome/ 1344

6 exp sleep/ or sleep quality/ 43995

7 exp Sleep Terrors/ 1

8 exp Narcolepsy/ 1662

9 sleep*.ti,ab. 87037

10 night awaking.ti,ab. 2

11 night waking.ti,ab. 236

12 waking at night.ti,ab. 32

13 night terror*.ti,ab. 284

14 bedtime.ti,ab. 2885

15 (insomnia or dyssomnia* or parasomnia* or hypersomnia or hypersomnolence or somnolence or apnea or circadian or wakefulness).ti,ab. 36588

16 (narcolepsy or narcoleptic*).ti,ab. 2548

17 (nocturnal adj2 myoclonus).ti,ab. 47

18 "Periodic Limb Movement".ti,ab. 254

19 (Willis adj2 Ekbom).ti,ab. 81

20 (Wittmaack adj2 Ekbom).ti,ab. 0

21 (restless adj2 leg*).ti,ab. 1946

22 tired*.ti,ab. 2932

23 or/4-22 115124

24 exp Health Disparities/ 11575

25 exp Socioeconomic Factors/ or exp Socioeconomic Status/ 98768

26 exp Ethnic Identity/ 19247

27 exp Racial Bias/ or exp Racial Identity/ or exp Racial Disparities/ 5248

28 exp at risk populations/ 41075

29 exp Minority Groups/ or exp Sexual Minority Groups/ 21181

30 exp "racial and ethnic groups"/ or asians/ or blacks/ or caribbean cultural groups/ or indigenous populations/ or "latinos/latinas"/ or "middle eastern and north african cultural groups"/ or multiracial/ or "people of color"/ 157817

31 exp Transgender/ 8211

32 exp "Culture (Anthropological)"/ 167731

33 exp Cultural Diversity/ 2520

34 exp Diversity/ 17538

35 exp Social Environments/ 193263

36 exp Neighborhoods/ or exp Home Environment/ 26870

37 exp Demographic Characteristics/ 203238

38 exp Social Connectedness/ 618

39 exp rural environments/ 22545

40 exp Urban Health/ or exp Urban Environments/ 29887

41 exp Suburban Environments/ 1158

42 exp Poverty Areas/ or exp Poverty/ 13501

43 exp Social Class/ 19465

44 exp "Uninsured (Health Insurance)"/ or exp Insurance/ 15434

45 exp Food Insecurity/ 1526

46 exp Educational Attainment Level/ 6326

47 exp "Income (Economic)"/ 7500

48 exp Racism/ or exp Systemic Racism/ 11103

49 exp Single Parents/ 3398

50 (ethnic* or Mexican American* or Hispanic or Latin*).ti,ab. 148394

51 (racial group* or Black* or African American* or Asian* or Native American* or Indigenous or Amish or Native Hawaiian or Arab* or Jews or Jewish or lgbtq or trangender* or gender minorit* or vulnerable population*).ti,ab. 195772

52 (cultur* or diversity or equity or inclusion).ti,ab. 464349

53 (neighborhood* or housing or unhoused or homeless* or marginaliz* or social vulnerabilit* or sociodemographic* or rural or suburban or urban or city or cities or segregation or redlining or playground* or connectedness).ti,ab. 263364

54 at risk population*.ti,ab. 2109

55 (green adj2 space*).ti,ab. 511

56 (recreation adj2 center*).ti,ab. 161

57 (home adj2 environment).ti,ab. 6058

58 (social adj2 environment).ti,ab. 11839

59 (socioeconomic* or inequalit* or economic* or poverty or social class*).ti,ab. 229680

60 Health Services Accessibility.ti,ab. 11

61 (care adj2 barrier*).ti,ab. 2404

62 access to care.ti,ab. 4418

63 access to resources.ti,ab. 2139

64 (food desert* or food apartheid or food insecurity).ti,ab. 2211

65 (educational adj2 status).ti,ab. 2532

66 (educational adj2 level).ti,ab. 11381

67 (parental adj2 education).ti,ab. 3499

68 income.ti,ab. 74428

69 racism.ti,ab. 15080

70 single parent*.ti,ab. 4212

71 single father*.ti,ab. 292

72 single mother*.ti,ab. 2408

73 or/24-72 1406355

74 exp Pediatrics/ 36902

75 (adolescen* or adolescent* child* or preschool* or boy* or girl* or teen* or youth* or juvenile* or pediatric* or paediatric*).ti,ab. 516832

76 or/74-75 528632

77 3 and 23 and 73 and 76 95

**CINAHL**

# Query Results

S87 S3 AND S29 AND S81 AND S86 115

S86 S82 OR S83 OR S84 OR S85 1,236,734

S85 TI ( adolescen* or adolescent* child* or preschool* or boy* or girl* or teen* or youth* or juvenile* or pediatric* or paediatric* ) OR AB ( adolescen* or adolescent* child* or preschool* or boy* or girl* or teen* or youth* or juvenile* or pediatric* or paediatric* ) 449,405

S84 (MH "Child+") 754,826

S83 (MH "Pediatrics+") 22,809

S82 (MH "Adolescence+") 603,315

S81 S30 OR S31 OR S32 OR S33 OR S34 OR S35 OR S36 OR S37 OR S38 OR S39 OR S40 OR S41 OR S42 OR S43 OR S44 OR S45 OR S46 OR S47 OR S48 OR S49 OR S50 OR S51 OR S52 OR S53 OR S54 OR S55 OR S56 OR S57 OR S58 OR S59 OR S60 OR S61 OR S62 OR S63 OR S64 OR S65 OR S66 OR S67 OR S68 OR S69 OR S70 OR S71 OR S72 OR S73 OR S74 OR S75 OR S76 OR S77 OR S78 OR S79 OR S80 1,487,551

S80 TI single father* OR AB single father* OR TI single mother* OR AB single mother* 951

S79 TI single parent* OR AB single parent* 1,410

S78 TI racism OR AB racism 6,443

S77 TI income OR AB income 82,577

S76 TI parental N2 education OR AB parental N2 education 2,734

S75 TI educational N2 level OR AB educational N2 level 11,249

S74 TI educational N2 status OR AB educational N2 status 3,483

S73 TI ( food desert* or food apartheid or food insecurity ) OR AB ( food desert* or food apartheid or food insecurity ) 5,017

S72 TI access to resources OR AB access to resources 936

S71 TI access to care OR AB access to care 7,695

S70 TI care N2 barrier* OR AB care N2 barrier* 7,695

S69 TI Health Services Accessibility OR AB Health Services Accessibility 63

S68 TI ( insurance or uninsured or medicaid ) OR AB ( insurance or uninsured or medicaid ) 73,489

S67 TI ( socioeconomic* or inequalit* or economic* or poverty or social class* ) OR AB ( socioeconomic* or inequalit* or economic* or poverty or social class* ) 165,772

S66 TI social N2 environment OR AB social N2 environment 6,467

S65 TI home N2 environment OR AB home N2 environment 5,110

S64 TI recreation N2 center* OR AB recreation N2 center* 124

S63 TI green N2 space* OR AB green N2 space* 641

S62 TI ( neighborhood* or housing or unhoused or homeless* or marginaliz* or social vulnerabilit* or sociodemographic* or rural or suburban or urban or city or cities or segregation or redlining or playground* ) OR AB ( neighborhood* or housing or unhoused or homeless* or marginaliz* or social vulnerabilit* or sociodemographic* or rural or suburban or urban or city or cities or segregation or redlining or playground* ) 238,684

S61 TI ( cultur* or diversity or equity or inclusion ) OR AB ( cultur* or diversity or equity or inclusion ) 327,283

S60 TI ( racial group* or Black* or African American* or Asian* or Native American* or Indigenous or Amish or Native Hawaiian or Arab* or Jews or Jewish or lgbtq or trangender* or gender minorit* or vulnerable population* ) OR AB ( racial group* or Black* or African American* or Asian* or Native American* or Indigenous or Amish or Native Hawaiian or Arab* or Jews or Jewish or lgbtq or trangender* or gender minorit* or vulnerable population* ) 152,792

S59 TI ( ethnic* or Mexican American* or Hispanic or Latin* ) OR AB ( ethnic* or Mexican American* or Hispanic or Latin* ) 115,890

S58 TI ( inequit* or disparit* or socioeconomic* ) OR AB ( inequit* or disparit* or socioeconomic* ) 98,238

S57 (MH "Single Parent") 1,978

S56 (MH "Systemic Racism") OR (MH "Racism+") 12,447

S55 (MH "Income+") 59,724

S54 (MH "Educational Status") 47,294

S53 (MH "Food Deserts") 34

S52 (MH "Medically Uninsured") 7,391

S51 (MH "Insurance+") 125,905

S50 (MH "Health Services Accessibility+") 107,376

S49 (MH "Social Class+") 14,848

S48 (MH "Poverty+") 30,818

S47 (MH "Economic Status") 1,203

S46 (MH "Economic Factors") 478

S45 (MH "Rural Population") OR (MH "Suburban Population") OR (MH "Urban Population") 20,112

S44 (MH "Sociodemographic Factors") OR (MH "Socioeconomic Factors+") 406,721

S43 (MH "Neighborhood Characteristics+") 758

S42 (MH "Home Environment") 12,584

S41 (MH "Social Environment+") 161,489

S40 (MH "Cultural Values+") 9,832

S39 (MH "Diversity, Equity, Inclusion") 754

S38 (MH "Cultural Diversity+") 17,012

S37 (MH "Culture+") 215,242

S36 (MH "LGBTQ+ Persons+") OR (MH "Transgender Persons+") OR (MH "Trans Women") OR (MH "Trans Men") 19,261

S35 (MH "Sexual and Gender Minorities+") 20,170

S34 (MH "Ethnic Groups+") OR (MH "Black Persons+") OR (MH "African Americans") OR (MH "Indigenous Peoples+") OR (MH "Jews+") 169,277

S33 (MH "Minority Groups") 14,327

S32 (MH "Ethnic Groups+") 169,277

S31 (MH "Healthcare Disparities") OR (MH "Socioeconomic Disparities in Health") OR (MH "Health Status Disparities+") 28,321

S30 (MH "Health Inequities") 1,366

S29 S4 OR S5 OR S6 OR S7 OR S8 OR S9 OR S10 OR S11 OR S12 OR S13 OR S14 OR S15 OR S16 OR S17 OR S18 OR S19 OR S20 OR S21 OR S22 OR S23 OR S24 OR S25 OR S26 OR S27 OR S28 109,975

S28 TI tired* OR AB tired 1,615

S27 TI Willis N2 Ekbom OR AB Willis N2 Ekbom OR TI Wittmaack N2 Ekbom OR AB Wittmaack N2 Ekbom OR TI restless N2 leg* OR AB restless N2 leg* 2,351

S26 TI Periodic Limb Movement OR AB Periodic Limb Movement 404

S25 TI nocturnal N2 myoclonus OR AB nocturnal N2 myoclonus 5

S24 TI ( narcolepsy or narcoleptic* ) OR AB ( narcolepsy or narcoleptic* ) 1,462

S23 TI ( insomnia or dyssomnia* or parasomnia* or hypersomnia or hypersomnolence or somnolence or apnea or circadian or wakefulness ) OR AB ( insomnia or dyssomnia* or parasomnia* or hypersomnia or hypersomnolence or somnolence or apnea or circadian or wakefulness ) 35,702

S22 TI bedtime OR AB bedtime 2,394

S21 TI night awaking OR AB night awaking OR TI night waking OR AB night waking OR TI waking at night OR AB waking at night OR TI night terror* OR AB night terror* 326

S20 TI sleep OR AB sleep 75,506

S19 (MH "Night Terrors") 107

S18 (MH "Narcolepsy") 1,301

S17 (MH "Sleep Stages+") 3,187

S16 (MH "Sleep Latency") 253

S15 (MH "Sleep Quality") 1,621

S14 (MH "Sleep Hygiene+") 2,627

S13 (MH "Sleep Duration") 263

S12 (MH "Sleep Deprivation") 4,282

S11 (MH "Sleep+") 33,150

S10 (MH "Apnea+") 18,129

S9 (MH "Restless Legs") 2,328

S8 (MH "Disorders of Excessive Somnolence+") 2,493

S7 (MH "Sleep Disorders, Circadian Rhythm+") 1,147

S6 (MH "Dyssomnias+") 33,500

S5 (MH "Sleep Disorders+") 45,803

S4 (MH "Sleep-Wake Transition Disorders+") 130

S3 S1 OR S2 37,571

S2 TI ( autism or autistic*or asperger* ) OR AB ( autism or autistic*or asperger* ) 30,294

S1 (MH "Autistic Disorder") OR (MH "Asperger Syndrome") 30,180

**Embase session results (18 Oct 2023)**

No. Query Results

#101 #4 AND #34 AND #96 AND #100 693

#100 #97 OR #98 OR #99 6346402

#99 adolescen* OR adolescent* OR child* OR preschool* OR boy* OR girl* OR teen* OR youth* OR juvenile* OR pediatric* OR paediatric*:ti,ab 5890444

#98 'pediatrics'/exp 139161

#97 'juvenile'/exp 4420566

#96 #35 OR #36 OR #37 OR #38 OR #39 OR #40 OR #41 OR #42 OR #43 OR #44 OR #45 OR #46 OR #47 OR #48 OR #49 OR #50 OR #51 OR #52 OR #53 OR #54 OR #55 OR #56 OR #57 OR #58 OR #59 OR #60 OR #61 OR #62 OR #63 OR #64 OR #65 OR #66 OR #67 OR #68 OR #69 OR #70 OR #71 OR #72 OR #73 OR #74 OR #75 OR #76 OR #77 OR #78 OR #79 OR #80 OR #81 OR #82 OR #83 OR #84 OR #85 OR #86 OR #87 OR #88 OR #89 OR #90 OR #91 OR #92 OR #93 OR #94 OR #95 9582862

#95 'health literacy':ti,ab 17818

#94 'media viewing':ti,ab 44

#93 'media watching':ti,ab 17

#92 'television watching':ti,ab 463

#91 'television viewing':ti,ab 1714

#90 'television viewing'/exp 4095

#89 'television in bedroom':ti,ab 6

#88 'media in bedroom':ti,ab 0

#87 'delayed diagnosis'/exp 18320

#86 'delayed intervention*' OR 'delayed diagnosis':ti,ab 16118

#85 'single mother*':ti,ab 1508

#84 'single father*':ti,ab 124

#83 'single parent*':ti,ab 3612

#82 racism:ti,ab 8992

#81 income*:ti,ab 223910

#80 'parental education'/exp 116

#79 'educational level*':ti,ab 32289

#78 'food desert*' OR 'food apartheid' OR 'food insecurity':ti,ab 10123

#77 'access to resources':ti,ab 2248

#76 'access to care'/exp 33

#75 'care barrier*':ti,ab 998

#74 insurance OR uninsured OR medicaid:ti,ab 341654

#73 socioeconomic* OR sociodemographic* OR inequalit* OR economic* OR poverty OR 'social class*':ti,ab 1393612

#72 'social environment*':ti,ab 14186

#71 'home environment*':ti,ab 9377

#70 'recreation center*':ti,ab 236

#69 'green space'/exp 159

#68 neighborhood* OR housing OR unhoused OR homeless* OR marginaliz* OR 'social vulnerabilit*' OR sociodemographic* OR rural OR suburban OR urban OR city OR cities OR segregation OR redlining OR playground* OR 'green space' OR connectedness:ti,ab 2199882

#67 cultur* OR diversity OR 'diversity, equity, inclusion':ti,ab 2671098

#66 'racial group*' OR black* OR 'african american*' OR asian* OR 'native american*' OR indigenous OR amish OR 'native hawaiian*' OR arab* OR jews OR jewish OR lgbtq OR trangender* OR 'gender minorit*' OR 'vulnerable population*':ti,ab 3139616

#65 ethnic* OR 'mexican american*' OR hispanic OR latin*:ti,ab 500532

#64 'at risk population*':ti,ab 9059

#63 inequit* OR disparit* OR socioeconomic*:ti,ab 370003

#62 'single-parent family'/exp 511

#61 'racism'/exp 14677

#60 'income'/mj 8431

#59 'educational status'/exp 140342

#58 'food desert'/exp 153

#57 'medically uninsured'/exp 8599

#56 'insurance'/exp 420539

#55 'health care access'/mj 13876

#54 'social status'/exp 247429

#53 'poverty'/exp 56829

#52 'economic aspect'/mj 20567

#51 'socioeconomics'/exp 1371121

#50 'urban population'/exp 53819

#49 'suburban population'/exp 942

#48 'rural population'/exp 58873

#47 'sociodemographics'/mj 1613

#46 'social vulnerability'/exp 683

#45 'social exclusion'/mj 680

#44 'neighborhood characteristic'/exp 438

#43 'social environment'/de 38214

#42 'cultural factor'/exp 68853

#41 'cultural diversity'/exp 3858

#40 'vulnerable population'/exp 28079

#39 'ancestry group'/exp 435131

#38 'ethnicity'/exp 120377

#37 'health care disparity'/exp 22611

#36 'socioeconomics'/de 169532

#35 'health disparity'/exp 35552

#34 #5 OR #6 OR #7 OR #8 OR #9 OR #10 OR #11 OR #12 OR #13 OR #14 OR #15 OR #16 OR #17 OR #18 OR #19 OR #20 OR #21 OR #22 OR #23 OR #24 OR #25 OR #26 OR #27 OR #28 OR #29 OR #30 OR #31 OR #32 OR #33 665694

#33 tired:ti,ab 4264

#32 'restless leg*':ti,ab 9257

#31 'wittmaack ekbom':ti,ab 3

#30 'willis ekbom':ti,ab 299

#29 'periodic limb movement':ti,ab 1046

#28 nocturnal NEAR/2 myoclonus 161

#27 narcolepsy OR narcoleptic*:ti,ab 12446

#26 insomnia OR dyssomnia* OR parasomnia* OR hypersomnia OR hypersomnolence OR somnolence OR apnea OR circadian OR wakefulness:ti,ab 428461

#25 bedtime*:ti,ab 11371

#24 night NEAR/3 terror* 450

#23 'waking at night':ti,ab 107

#22 'night waking':ti,ab 498

#21 'night awaking':ti,ab 9

#20 sleep*:ti,ab 355943

#19 'periodic limb movement disorder'/exp 3243

#18 'narcolepsy'/exp 10637

#17 'sleep medicine'/exp 5184

#16 'sleep stage'/exp 48579

#15 'sleep latency'/exp 6023

#14 'sleep quality'/exp 38686

#13 'sleep hygiene'/exp 2804

#12 'sleep time'/exp 40338

#11 'sleep deprivation'/exp 20960

#10 'sleep'/mj 56628

#9 'sleep apnea syndromes'/exp 102801

#8 'restless legs syndrome'/exp 12074

#7 'disorders of excessive somnolence'/exp 15935

#6 'circadian rhythm'/exp 104225

#5 'sleep disorder'/exp 341346

#4 #1 OR #2 OR #3 64037

#3 autistic*:ti,ab 21862

#2 asperger*:ti,ab 3065

#1 'autism'/mj 56785
